# Sex-based differences in myocardial infarction-induced kidney damage following cigarette smoking exposure: more renal protection in premenopausal female mice

**DOI:** 10.1042/BSR20193229

**Published:** 2020-06-23

**Authors:** Nada J. Habeichi, Ali Mroueh, Abdullah Kaplan, Rana Ghali, Hiam Al-Awassi, Cynthia Tannous, Ahmad Husari, Abdo Jurjus, Raffaele Altara, George W. Booz, Ahmed El-Yazbi, Fouad A. Zouein

**Affiliations:** 1 Department of Pharmacology and Toxicology, American University of Beirut Faculty of Medicine, Beirut, Lebanon; 2 INSERM Department of Signaling and Cardiovascular Pathophysiology-UMR-S1180, University Paris-Saclay, Châtenay-Malabry, France; 3 Department of Internal Medicine, Respiratory Diseases and Sleep Medicine, American University of Beirut Medical Center, Beirut, Lebanon; 4 Department of Anatomy, Cell Biology, and Physiology, American University of Beirut Medical Center, Beirut, Lebanon; 5 Institute for Experimental Medical Research, Oslo University Hospital and University of Oslo, Oslo, Norway; 6 KG Jebsen Center for Cardiac Research, Oslo, Norway; 7 Department of Pathology and Toxicology, School of Medicine, University of Mississippi Medical Center, Jackson, MS, U.S.A.; 8 Department of Pharmacology and Toxicology, School of Medicine, University of Mississippi Medical Center, Jackson, MS, U.S.A.; 9 Department of Pharmacology and Toxicology, Faculty of Pharmacy, Alexandria University, Alexandria, Egypt

**Keywords:** Cigarette smoking, gender differences, Kidney damage, Myocardial infarction

## Abstract

The impact of cigarette smoking (CS) on kidney homeostasis in the presence of myocardial infarction (MI) in both males and females remains poorly elucidated. C57BL6/J mice were exposed to 2 weeks of CS prior to MI induction followed by 1 week of CS exposure in order to investigate the impact of CS on kidney damage in the presence of MI. Cardiac hemodynamic analysis revealed a significant decrease in ejection fraction (EF) in CS-exposed MI male mice when compared with the relative female subjects, whereas cardiac output (CO) comparably decreased in CS-exposed MI mice of both sexes. Kidney structural alterations, including glomerular retraction, proximal convoluted tubule (PCT) cross-sectional area, and total renal fibrosis were more pronounced in CS-exposed MI male mice when compared with the relative female group. Although renal reactive oxygen species (ROS) generation and glomerular DNA fragmentation significantly increased to the same extent in CS-exposed MI mice of both sexes, alpha-smooth muscle actin (α-SMA) and connective tissue growth factor (CTGF) significantly increased in CS-exposed MI male mice, only. Metabolically, nicotinamide phosphoribosyltransferase (NAMPT) and nicotinamide riboside-1 (NMRK-1) substantially increased in CS-exposed MI female mice only, whereas sirtuin (SIRT)-1 and SIRT-3 substantially decreased in CS-exposed MI male mice compared with their relative female group. Additionally, renal NAD levels significantly decreased only in CS-exposed MI male mice. In conclusion, MI female mice exhibited pronounced renal protection following CS when compared with the relative male groups.

## Introduction

Cardiovascular diseases (CVDs) remain the leading cause of death in males and females worldwide [1,2]. The World Health Organization (WHO) estimates that more than 17 million CVD deaths annually are due to ischemic heart diseases, involving mostly myocardial infarction (MI) and strokes [3] (http://www.who.int/cardiovascular_diseases/en/). According to the 2016 heart disease and stroke statistics, one American will suffer an acute MI every 42 s around the clock [4]. The prevalence of MI, however, is lower in females when compared with age-matched males, but picks up with aging to reach comparable levels following menopause [5,6]. For instance, the male to female MI risk ratio under 45 years of age is 10:1, while dropping substantially in those over 75 years of age to 2:1 [7]. Noteworthy, patients diagnosed with MI are not only at high risk of heart failure development, but also of multiple comorbidities including a 29-fold increase in acute kidney injury (AKI) through the cardio-renal interrelationship [8,9]. In fact, MI-induced cardiac systolic dysfunction leads to a decrease in stroke volume and cardiac output (CO) which hypoperfuses the kidney, markedly enhancing the risk of kidney damage [10–12]. Multiple factors including hypertension, diabetes mellitus, and cigarette smoking (CS) are identified as high risk factors for both MI and AKI development [13].

Recent published evidence positively correlates CS to AKI onset and progression via both direct and indirect mechanisms [14–16]. CS contains approximately 5000 potential toxic chemicals including, among others, oxidative gases, free radicals, nicotine, and amide [17]. These chemicals have been shown to enhance reactive oxygen species (ROS) production, mitochondrial dysfunction, fibrosis, and cell death, all of which are hallmark features of AKI development [18–24]. Besides all the undesired chemicals in CS and their harmful effects, nicotine itself is known to induce apoptosis in podocytes and fibrosis in proximal convoluted tubules (PCTs), promoting therefore kidney damage [25,26].

Interestingly, pre-menopausal women are less prone to develop AKI following CS exposure when compared with age matched men [27]. This sexual dimorphism is attributed to the well-known renal protective effects of endogenous estrogen (E2) that is reported in multiple pre-clinical and clinical studies [28,29]. For instance, E2 has been tightly linked to reduced kidney injury following renal ischemia reperfusion through decreasing glomerular mesangial cells damage, albuminuria, and tubulointerstitial fibrosis [30,31]. In contrast, estrogen receptor-α knockout is linked to enhanced podocyte apoptosis in female mice, resulting in extracellular matrix (ECM) accumulation and glomerular hypertrophy, increasing consequently albuminuria and kidney injury [32]. To date, very little is known about the impact of CS on males and female’s kidneys in the presence of MI. This is the first study to provide experimental evidence of sex discrepancy between CS-exposed male and female mice with MI, suggesting therefore the potential importance of sex hormones in modulating the pathophysiological responses of this disease model.

## Materials and methods

### Animal use

All animal experiments in the present study were conducted according to the experimental protocols approved by American University of Beirut Animal Care and Use Committees (IACUC # 18-2-RN560) in compliance with the National Institutes of Health Guide for the Care and Use of Laboratory Animals, 8th edition [33]. Age-matched male and female C57BL6/J mice (5 months old) were maintained in the animal care facility at the American University of Beirut Medical Center under optimum conditions with 12 light/12 dark hours cycle with unlimited access to water and standard chow.

### Experimental design

Five months old C57BL6/J male and premenopausal female mice were allocated into four groups: control group (3 weeks of air exposure) (*n* = 6), CS exposure (non-MI) group (3 weeks of CS exposure) (*n* = 6), MI group (*n* = 6), and MI+CS group (*n* = 10) (2 weeks of CS exposure followed by MI and another week of CS exposure). Seven days post-MI, MI male and female mice were killed and at the end of week 3, control, CS-exposed (non-MI), and CS-exposed MI mice of both sexes were killed. One hundred microliters heparin (Heparin Sodium 1000 IU/ml) was administrated to mice 30 min prior to sacrifice. Following deep anesthesia with 4% isoflurane, blood was collected by cardiac puncture, centrifuged for 10 min at 2200 rpm and the plasma flash-frozen in liquid nitrogen and stored at −80°C. Mice were then subjected to cervical dislocation. For kidney collection, right kidney was harvested and immediately placed into a cryotube in liquid nitrogen followed by storage at −80°C for molecular work. Left kidney was harvested into 4% zinc formalin tubes for histology. A portion was also used to assess ROS (see below).

### CS exposure protocol

Conscious C57BL6/J aged matched male and female mice were retained and exposed to mainstream tobacco cigarette smoke using nose only exposure apparatus (ONARES, CH Technologies, U.S.A.). 3R4F scientific cigarettes (University of Kentucky, Lexington, KY, U.S.A.), scientifically prepared cigarettes concentrated with toxins, were used and placed into the cigarette puffer to generate puffs at a constant frequency of one puff/50 s, a volume of 2 ml/puff, and a duration of 2.5 s/puff allowing a total particle matter (TPM) concentration of approximately 100 mg/cm^3^/mouse/session, 9.4 mg tar, and 0.726 mg nicotine per cigarette. Mice were exposed to two 90-min sessions daily (10 cigarettes/session), 7 days/week either for 3 weeks or for 2 weeks before inducing MI, then one additional week thereafter.

### Myocardial infarction

MI was induced by left anterior descending (LAD) coronary artery ligation. Heart rate, body temperature, and respiratory rate were assessed in order to reduce any surgical complications. The mouse was placed on a heating pad to prevent anesthesia-induced hypothermia. Fifteen min prior to the surgery, tramadol (0.05–0.1 mg/kg i.p.) and isoflurane (2–3% in oxygen) inhalation were given to induce analgesia and general anesthesia. Orotracheal intubation was performed to maintain normal respiratory rate by placing a needle into the trachea and connecting it to a mini automated ventilator (Harvard Apparatus). LAD coronary artery, left ventricle, and left atrium were exposed by excising between the ribs of the left thorax. MI was induced by LAD ligation with 7-0 polypropylene suture at 1–3 mm underneath the left atrium appendage. Successful MI induction was confirmed by the blanching of the tissue downstream of the ligation site, by ECG, and by echocardiography 24 h after surgery. Immediately following successful LAD ligation, the chest was closed and mice were placed on a warm pad for recovery. Once they groomed freely, MI mice were caged back individually and monitored on daily basis for full recovery.

### Echocardiography

Vevo 2100™ High-Resolution Imaging System (Visual Sonics, Toronto, Canada) was used to perform echocardiography according to the American Society of Echocardiography guidelines. In order to induce general anesthesia, mice were exposed to isoflurane (1.5% in oxygen) inhalation. Body temperature was maintained at 37°C using rectal thermostat and warming plate. The transducer was then placed on the left thorax and ultrasound beam was directed at the mid papillary muscle level in order to obtain M-mode and B-mode echocardiography images, in the parasternal long- and short-axis views. Ejection fraction (EF) and CO were measured at baseline, at the end of week 2 (before the induction of MI), and at the end of week 3 (before killed).

### Hematoxylin and eosin staining

Hematoxylin and eosin (H&E) staining was used to highlight glomerular retraction for all experimental groups. Four micrometers thick renal sections from each mouse were stained with H&E according to standard laboratory protocol. Briefly, kidney tissues were first fixed in 4% formalin, then dehydrated and embedded in paraffin. Dewaxing and hydration steps of paraffin-embedded kidneys were performed, and then the tissue was stained with H&E and examined under light microscope with 40× magnification. Glomeruli that were sectioned at midpoint with commonly visible afferent and efferent arterioles were included in the analysis. The ones that had deformation in shape and borders and were sectioned close to the pole of the glomerular sphere were excluded.

### Periodic acid Schiff staining

Four μm thick kidney sections from each mouse were stained with periodic acid Schiff (PAS) according to standard laboratory protocol to assess PCT dilatation. Briefly, paraffin embedded kidneys underwent dewaxing and hydration steps. Dehydration with increasing percentages of ethanol (75, 95% and 100%) was performed, kidney tissues were then stained with 0.5% PAS for 10 min, followed by Schiff reagent for 10–20 min, and washed for 5 min. Finally, slides were mounted using mounting medium and observed under light microscope at 40×. PCT cross-sectional area was measured using Image J software (https://imagej.nih.gov/ij/)

### Masson’s trichrome staining

Masson’s trichrome (MTC) staining was used to assess total renal fibrosis. Paraffin embedded kidneys underwent dewaxing and hydration steps, soaked in Bouin solution for one h at 56°C, then washed and rinsed in distilled water. Kidney tissues were incubated for 10 min with hematoxylin, then washed and stained in biebrich scarlet acid fuchsin. After washing, kidney sections were differentiated in phosphomolybdic-phosphotungstic acid solution for 10 min, transferred to aniline blue solution, stained for 5 min and observed under light microscopy at 10× magnification. Total renal fibrosis was measured using Image J software (https://imagej.nih.gov/ij/). Images were converted to gray scale followed by threshold modification in order to measured red-stained fibrosis.

### Dihydroethidium staining

Total ROS score was assessed using dihydroethidium (DHE) staining (Calbiochem, Darmstadt, Germany) and performed on whole kidney tissue sections. Ten µM DHE was used to incubate unstained sections under dark condition in a humidified chamber at room temperature for 45 min. Images of kidney tissues were acquired using Laser Scanning Fluorescent Microscope (Zeiss Axio) under 20× magnification.

### Terminal deoxynucleotidyl transferase dUTP nick end labeling

Glomerular DNA fragmentation was evaluated using Click-It Plus TUNEL Assay (Invitrogen). Briefly, kidney tissues underwent dewaxing and rehydration steps, then DNA fragmentation detection kit was used to perform terminal deoxynucleotidyl transferase dUTP nick end labeling (TUNEL) staining as described by manufacturer’s instructions. Laser Scanning Fluorescent Microscope (Zeiss Axio) was used to assess glomerular DNA fragmentation under 40× magnifications. Glomeruli with green nuclear labeling were considered as TUNEL positive.

### Immunofluorescence

The protein expression level of alpha-smooth muscle actin (α-SMA) in kidney tissues was evaluated using immunofluorescence (IF). Kidney slides were placed in an antigen retrieval buffer at 95°C for 15 min, followed by two washes with tris-buffered saline (TBS)-Triton 0.025%, 5 min each. To block non-specific binding of α-SMA antibody, slides were incubated with 10% normal goat serum (NGS) and with 1% bovine serum albumin (BSA) in TBS for 2 h at room temperature. Kidney slides were then incubated with a dilution of α-SMA antibody (Abcam, ab5694), in TBS (1:200) at 4°C overnight, and washed twice with TBS-T 0.025%, 5 min each. Incubation with a dilution of secondary antibody (FITC) (Abcam, ab97050) in TBS (1:100) for 1 h was performed, followed by two washes with TBS, 5 min each. Dapi was then added, and slides were washed twice with phosphate-buffered saline (PBS), 5 min each. Laser Scanning Fluorescent Microscope (Zeiss Axio) was used to detect renal α-SMA protein expression under 20× magnification.

### RNA extraction and RT-qPCR

Snap frozen kidney tissues were crushed under liquid nitrogen, then total RNA was extracted using Trizol according to manufacturer’s instructions (Thermo Fisher Scientific, Grand Island, NY, U.S.A.). RNA was quantified using NanoDrop ND-1000 UV–Vis Spectrophotometer. Differences in mRNA expression levels were quantified using real time q-PCR. During reverse transcriptase (RT) steps, a c-DNA synthesis kit (QuantiTect Reverse Transcription Kit, Qiagen) was used to synthesize c-DNA from 1 μg RNA, followed by real time PCR analysis in a CFX96 real-time PCR system (Bio-Rad, Germany). Afterward, the synthesized c-DNA was loaded in duplicate with 0.05 µl of the forward and reverse primers of the following genes: nicotinamide phosphoribosyltransferase (NAMPT), nicotinamide riboside kinase-1 (NMRK1), sirtuin-1 (SIRT-1), sirtuin-3 (SIRT-3), poly [ADP-ribose] polymerase (PARP-1), connective tissue growth factor (CTGF), and α-SMA and mixed with SYBR® Green for qPCR steps. To normalize gene expression between different samples, glyceraldehyde-3-phosphate dehydrogenase (GAPDH) was used. No-template (water without DNA) was used to check for non-specific amplification. Normalized fold expression relative to the control was calculated and plotted by Biorad CFX-manager to compare differential gene expression. Primer sequences were used as listed in Table 1

**Table 1:** List of primers used in the present study

Primer	Forward	Reverse
**GAPDH**	TGTGTCCGTGGATCTGA	TTGCTGTTGAAGTCGCAGGAG
**NAMPT**	ACCAGCGGGGAACTTTGTTA	ACATAACAACCCGGCCACAT
**NMRK-1**	CTTGAAGCTTGCTCTGCGAC	GTGTCGTCTTCCCTCCGTTT
**SIRT-1**	CGGCTACCGAGGTCCATATAC	ACAATCTGCCACAGCGTCAT
**SIRT-3**	GATTCGGATGGCGCTTGAC	TCTCCCACCTFTAACACTCCC
**PARP-1**	ACACCACAAAACCTCAGCCA	ACAAACCACAAACAACCGGC
**α-SMA**	CAGCGGGCATCCACGAAA	GGCCCAGCTTCGTCGTATT
**CTGF**	ACCCAACTATGATGCGAGCC	GGTAACTCGGGTGGAGATGC

### Enzyme-linked immunosorbent assay

#### Assessment of plasma TNF-α levels

Enzyme-linked immunosorbent assay (ELISA) Stemcell Technologies kit (catalog #02030) was used for the detection and measurement of mouse TNF-α in plasma. Plasma samples were diluted 1:1 in ELISA diluent for a total minimum volume of 250 μl per dilution. One hundred microliters of diluted samples were then loaded in duplicate in ELISA strip plates pre-coated with the cytokine of interest and incubated at room temperature for 2 h. Afterward, each well was washed five times with 300 μl washing buffer. One hundred microliters/well of diluent detection antibody was added and the plate was incubated at room temperature for 1 h. Each well was then washed five times with 300 μl washing buffer and 100 μl/well of diluted streptavidin horseradish peroxidase (SA-HRP) was added and the plate was incubated at room temperature for 1 h. After washing steps, 100 μl/well of 3,3′,5,5′-tetramethylbenzidine (TMB) substrate was added to all wells and the plate was incubated for 15 min at room temperature. One hundred microliters/well of stopping solution was then added to each well and the absorbance was measured at 450 nm in a microplate reader.

#### Assessment of plasma 17-β estradiol levels

ELISA Abcam kit (catalog#108667) was used for the detection and measurement of baseline 17-β estradiol in plasma, to verify that all female mice are in the premenopausal phase [34] (Table 2). Twenty-five microliters of samples were loaded in duplicate, 17-β Estradiol-HRP conjugated was then added, and the plate was incubated at room temperature for 2 h. Afterward, each well was washed three times with 300 μl washing buffer. After washing steps, 100 μl/well of TMB substrate was added to all wells and the plate was incubated for 30 min at room temperature. One hundred microliters/well of stopping solution was then added to each well and the absorbance was measured at 450 nm in a microplate reader.

**Table 2:** Baseline plasma estrogen levels comparison between female mice groups in the presence of CS and MI

Female mice group	Mean ± SEM (pg/ml)	Mice/group	*P* value
**Ctrl**	31.678 ± 6.051	3	0.5051
**CS**	38.211 ±2.646	5	
**MI**	34.106 ± 2.045	5	
**MICS**	35.412 ± 1.907	5	

Data were analyzed for significance using one-way ANOVA. All bars represent mean ± SEM (*n* = 3–5); CS: cigarette smoking; Ctrl: Control; MI: myocardial infarction.

#### Total NAD extraction and quantification

Twenty milligrams of kidney tissues were extracted using 75% ethanol and 25% HEPES (10 mM pH 7.1) and diluted 1:20 in distilled water for a total minimum volume of 50 µl per dilution to reach a concentration within the standard curve. Twenty five microliters of NAD samples were then loaded in duplicate in 96-well microtiter-plate. Afterward, 100 µl/well of reaction buffer [600 mM ethanol, 0.5 mM 3-(4.5dimethylthiazol-2-yl)-2.5- diphenyltetrazolium bromide (MTT), 2 mM phenazine ethosulfate (PES), 120 mM Bicine (pH7.8), yeast alcohol dehydrogenase (SIGMA A3263 > 300 µ/mg)] was added to the extract. The kinetic of the reaction (OD at 550 nm, every 30 s for 20 min) was followed up on LB 942 multimode reader and NAD was then quantified using linear regression curve equation method between NAD standard concentration and the slope of the regression line.

### Statistical analysis

To perform statistical analysis, Graphpad Prism7 software was used. Statistical comparisons were performed using one-way ANOVA or two-way ANOVA and Sidak’s multiple comparisons test. Results were expressed as mean ± standard error of the mean (SEM). A *P*-value of *P*<0.05 (*) was considered significant.

## Results

### CS exposure decreased EF but not CO in MI male mice when compared with the relative female subjects

**Figure 1:**
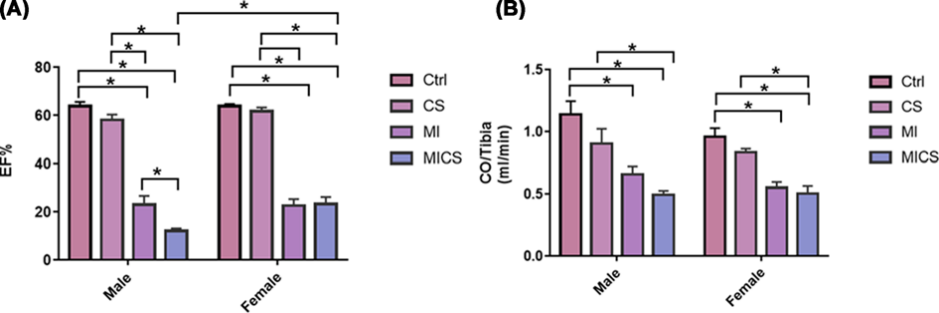
EF and CO comparison between male and female mice following CS exposure in the presence of MI Cardiac functional assessment. EF markedly decreased in CS-exposed MI males when compared with their relative female subject (**A**). (**B**) Co significantly decreased in CS-exposed MI mice of both sexes**.** Data were analyzed for significance using two-way ANOVA. All bars represent mean ± SEM (*P value < 0.05; *n* = 5–9); CS: cigarette smoking; Ctrl: Control; MI: myocardial infarction.

CS-exposed MI mice of both sexes experienced a significant decrease in EF when compared with non-exposed and CS-exposed (non-MI) mice. CS-exposed MI male mice exhibited a marked decrease in EF when compared with the MI group. No significant change in CS-exposed MI female mice in EF when compared with MI female mice, however, was observed. CS-exposed (non-MI) mice of both sexes showed no significant change in EF when compared with their relative non-exposed groups, whereas MI mice of both sexes experienced a substantial decrease in EF when compared with non-exposed and CS-exposed (non-MI) groups. Cross-sex analysis revealed a marked decrease in EF in CS-exposed MI males when compared with the relative female group. No significant difference between control male and female mice was observed (Figure 1A). As for the impact of CS and MI on CO, CS-exposed MI mice of both sexes experienced a comparable decrease in CO when compared with non-exposed and CS-exposed (non-MI) groups. CS-exposed (non-MI) male and female groups, on the other hand, showed no significant change in CO when compared with their relative non-exposed groups. MI mice of both sexes, however, exhibited a substantial comparable decrease in CO when compared with non-exposed groups. No significant difference between control male and female mice was observed (Figure 1B).

### Plasma TNF-α was undetectable in all mice groups of both sexes

The effect of CS and MI on systemic inflammation was assessed in terms of plasma TNF-α levels. Plasma TNF-α was measured using Stem Cells Technologies TNF-α ELISA kit. Plasma TNF-α protein levels were undetectable in all groups of both sexes in the presence and absence of CS and MI.

### CS exposure increased glomerular retraction in MI male mice compared with the relative female subjects

**Figure 2:**
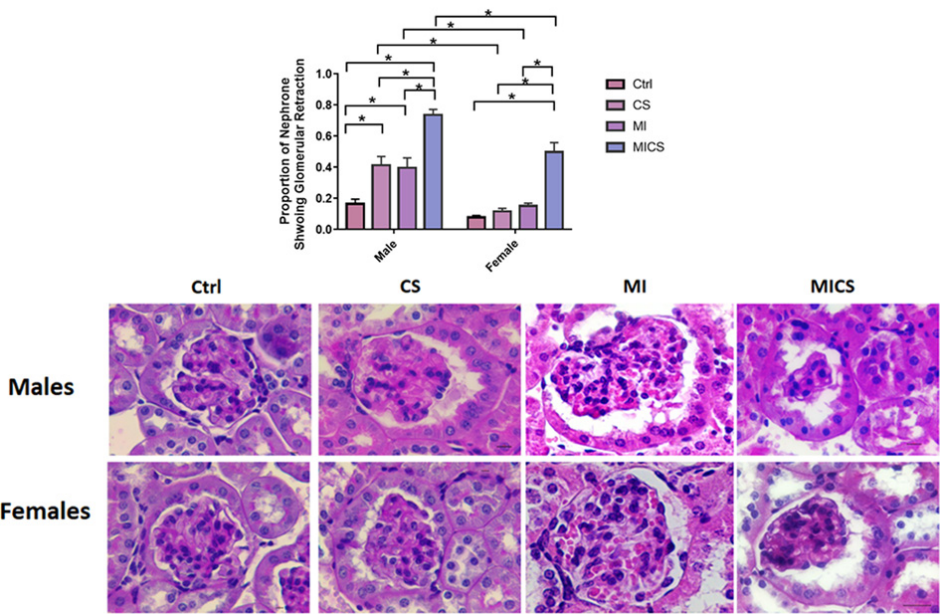
Glomerular retraction comparison between male and female mice following CS exposure in the presence of MI Enhanced glomerular retraction was observed in CS-exposed MI males when compared with the relative female subject. Representative image of each H&E stained group is shown. Data were analyzed for significance using two-way ANOVA. All bars represent mean ± SEM (*P value < 0.05; *n* = 3–9); CS: cigarette smoking; Ctrl: Control; MI: myocardial infarction.

Glomerular retraction was assessed by H&E staining. Figure 2 shows a significant increase in glomerular retraction in CS-exposed MI mice of both sexes compared with the relative non-exposed, CS-exposed (non-MI), and MI groups. CS-exposed (non-MI) and MI male mice experienced a marked increase in glomerular retraction when compared with non-exposed groups. However, no significant change in glomerular retraction in CS-exposed (non-MI) and MI female mice when compared with the non-exposed group was observed. Cross-sex analysis revealed a substantial increase in glomerular retraction in CS-exposed (non-MI), MI, and CS-exposed MI male mice when compared with the relative female groups. No significant difference between control male and control female mice was observed.

### CS exposure increased PCT cross-sectional area in MI male mice compared with the relative female subjects

**Figure 3:**
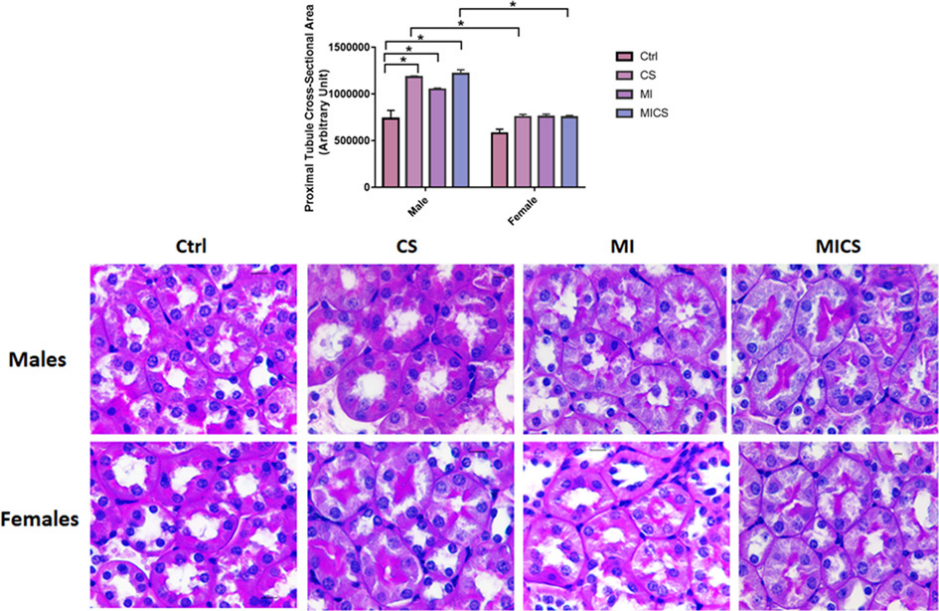
CS exposure increased PCT cross-sectional area in MI male mice compared to the relative female subjects PCT dilatation was substantially increased in CS-exposed MI males when compared with the relative female subjects. Representative image of each PAS stained group is shown. Data were analyzed for significance using two-way ANOVA. All bars represent mean ± SEM (*P value < 0.05; *n* = 3–8); CS: cigarette smoking; Ctrl: Control; MI: myocardial infarction.

PCT cross-sectional area was assessed by PAS staining. Figure 3 reveals an enhanced PCT cross-sectional area in CS-exposed (non-MI), MI, and CS-exposed MI male mice when compared with the non-exposed group. All female mouse groups, however, exhibited no significant change in PCT dilatation. Cross-sex analysis revealed a substantial increase in PCT cross-sectional area in CS-exposed (non-MI), and CS-exposed MI male mice when compared with the relative female groups. No significant difference between control male and female mice was observed.

### CS exposure increased total renal fibrosis but not α-SMA and CTGF in MI male mice compared with the relative female subjects

**Figure 4:**
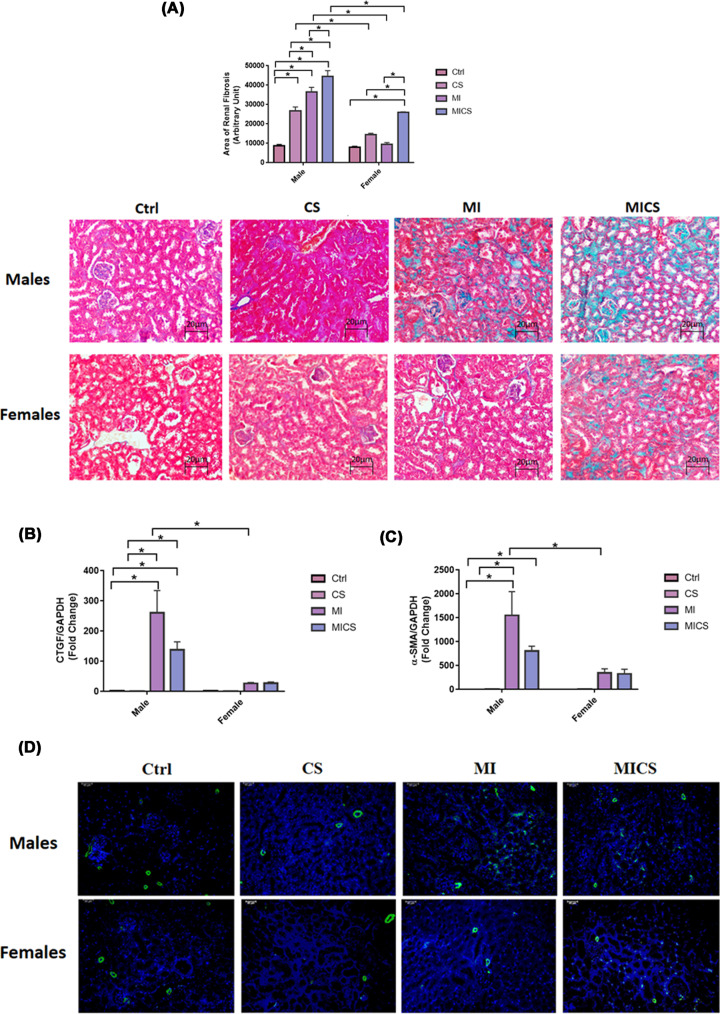
CS exposure increased total renal fibrosis but not α-SMA and CTGF in MI male mice compared to the relative female subjects Total renal fibrosis significantly increased in CS-exposed MI males when compared with the relative female subjects (**A**). Representative image of each MTC stained group is shown. α-SMA and CTGF significantly increased in CS-exposed MI male mice, only (**B,C**). (**D**) α-SMA accumulation in the peritubular space of CS-exposed MI male mice, only. Representative image of each IF stained group is shown. Data were analyzed for significance using two-way ANOVA. All bars represent mean ± SEM (*P value < 0.05; *n* = 3–8); CS: cigarette smoking; Ctrl: Control; MI: myocardial infarction.

Total renal fibrosis was assessed by MTC staining. Figure 4A reveals a significant increase in total renal fibrosis in CS-exposed MI mice of both sexes when compared with the relative non-exposed, CS-exposed (non-MI), and MI groups. A marked increase in total renal fibrosis in CS-exposed (non-MI) mice when compared with the non-CS exposed group was only seen in male mice. Additionally, MI male mice experienced a significant increase in total renal fibrosis when compared with non-exposed and CS-exposed groups. Cross-sex analysis revealed a significant increase in total renal fibrosis in CS-exposed (non-MI), MI, and CS-exposed MI male mice when compared with the relative female mice. No significant difference between control male and female mice was observed. Figure 4B shows a marked increase in renal CTGF mRNA expression levels in MI and CS-exposed MI male mice when compared with non-exposed and CS-exposed mice. No significant change in renal CTGF mRNA expression levels in all female mice, however, was observed. Cross-sex analysis revealed a substantial increase in renal CTGF mRNA expression levels in MI male mice when compared with the relative male group. Figure 4C shows a significant increase in renal α-SMA mRNA expression levels in CS-exposed MI and MI male mice when compared with non-exposed groups. MI male mice experienced a significant increase in renal α-SMA mRNA expression levels when compared with CS-exposed mice. No significant change in all female mice, however, was observed. Cross-sex analysis revealed a substantial increase in renal α-SMA mRNA expression levels in MI male mice when compared with the relative female group. Our finding is further fortified with IF data showing an accumulation of α-SMA in the peritubular space in MI and CS-exposed MI male mice, only (Figure 4D)**.**

### CS exposure increased total ROS score and glomerular DNA fragmentation in MI mice of both sexes

**Figure 5:**
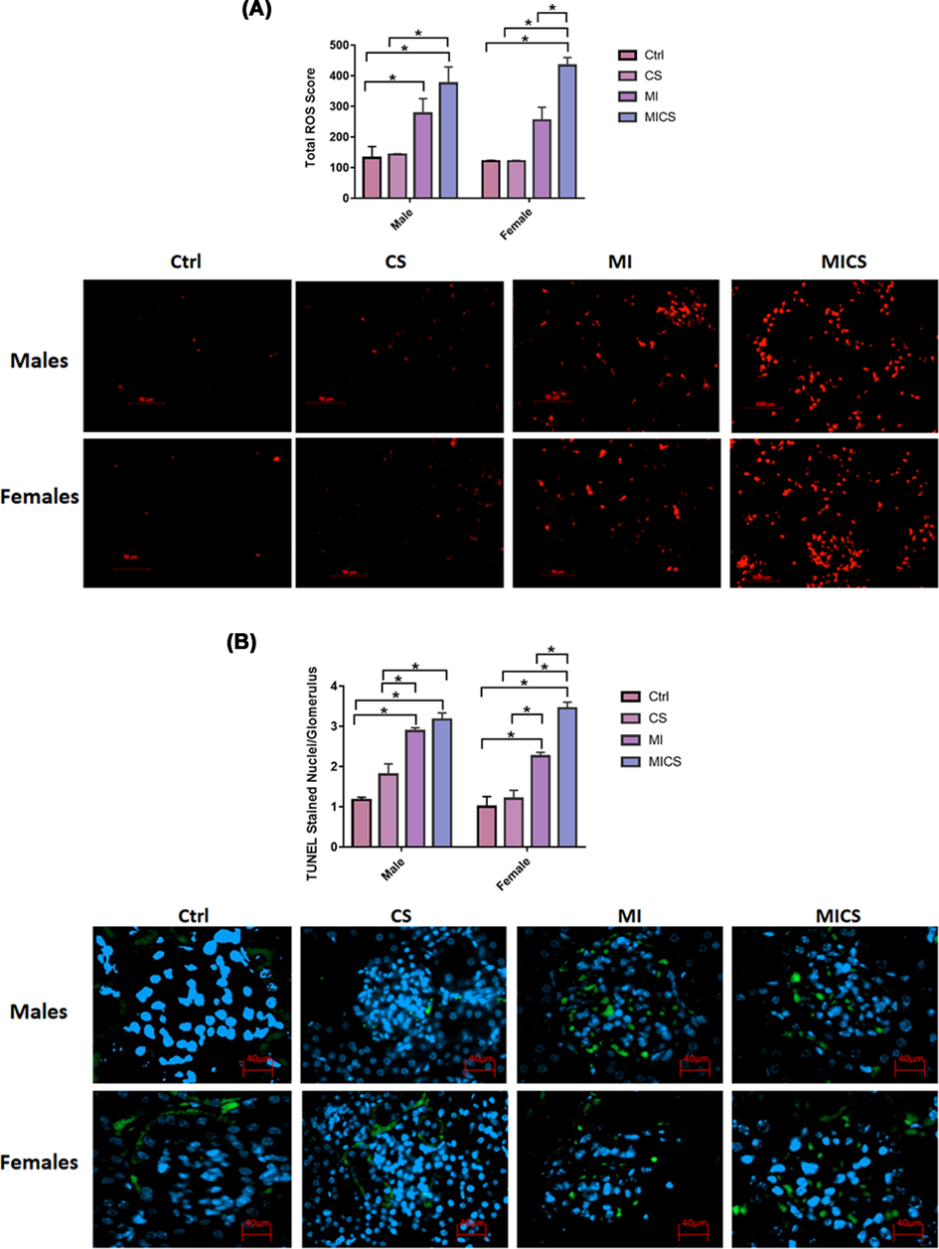
Total ROS score and glomerular DNA fragmentation comparison between male and female mice following CS exposure in the presence of MI Total ROS score (**A**) and glomerular DNA fragmentation (**B**) significantly increased in CS-exposed MI mice of both sexes. Representative image of each DHE and TUNEL stained group is shown. Data were analyzed for significance using two-way ANOVA. All bars represent mean ± SEM (*P value < 0.05; *n* = 3–5); CS: cigarette smoking; Ctrl: Control MI: myocardial infarction.

Figure 5A shows a significant and comparable increase in total ROS in CS-exposed MI mice of both sexes when compared with non-exposed and CS-exposed (non-MI) groups. A significant increase in total ROS score in CS-exposed MI mice when compared with the MI group was observed in female mice, only. CS-exposed (non-MI) male and female mice, however, showed no significant change in total ROS score when compared with their relative non-exposed groups. A marked increase in total ROS score in MI mice when compared with the non-exposed group was observed in male mice, only. No significant difference between control male and female mice was observed. Figure 5B shows a comparable significant increase in glomerular DNA fragmentation in CS-exposed MI mice of both sexes when compared with non-exposed and CS-exposed (non-MI) mice. A significant increase in glomerular DNA fragmentation in CS-exposed MI mice when compared with the MI group was observed in female mice, only. CS-exposed (non-MI) male and female mice, however, showed no significant change in glomerular DNA fragmentation when compared with their relative non-exposed groups. A marked increase in glomerular DNA fragmentation in MI mice when compared with the non-exposed group and CS-exposed groups was observed in mice of both sexes. No significant difference between control male and female mice was observed.

### CS exposure altered NAD pathway enzymes in MI mice of both sexes

**Figure 6:**
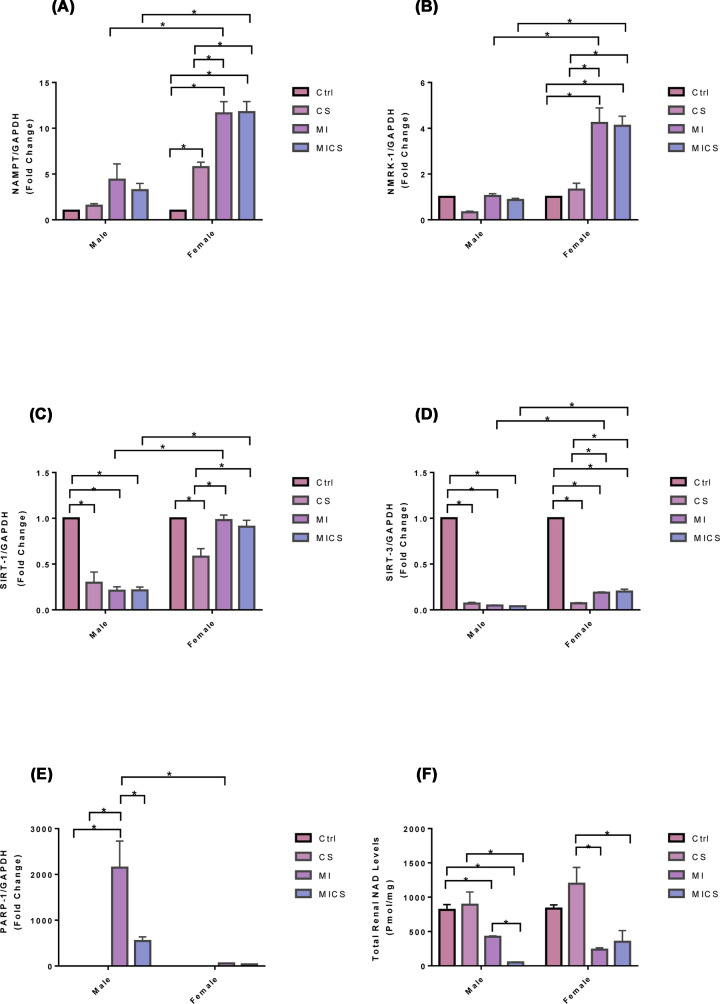
NAD biosynthetic, NAD dependent, total renal NAD levels comparison between male and female mice following CS exposure in the presence of MI NAMPT and NMRK-1 mRNA expression levels significantly increased in CS-exposed MI female mice when compared with the relative male subjects (**A,B**). SIRT-1 and SIRT-3 mRNA expression levels markedly decreased in CS-exposed MI male mice when compared with the relative female subjects (**C,D**). PARP-1 mRNA expression levels and total renal NAD levels substantially decreased in CS-exposed MI male mice when compared with the MI group (**E,F**). Data were analyzed for significance using two-way ANOVA. All bars represent mean ± SEM (*P value < 0.05; *n* = 3–10).

Figure 6A shows a significant increase in renal NAMPT mRNA expression levels in CS-exposed MI female mice when compared with non-exposed and CS-exposed (non-MI) mice. CS-exposed (non-MI) female mice experienced a marked increase in NAMPT mRNA expression levels when compared with non-exposed mice. Coherently, MI female mice showed a substantial increase in NAMPT mRNA expression levels when compared with non-exposed and CS-exposed (non-MI) groups. No significant change in NAMPT mRNA expression levels, however, in all male mice was observed. Cross-sex analysis revealed a significant increase in NAMPT mRNA expression levels in MI and CS-exposed MI female groups when compared with the relative male mice. Figure 6B reveals a marked increase in renal NMRK-1 mRNA expression levels in CS-exposed MI female mice when compared with non-exposed and CS-exposed (non-MI) female groups. No significant change in renal NMRK-1 mRNA expression levels in CS-exposed (non-MI) mice when compared with non-exposed mice of both sexes, however, was observed. A marked increase in renal NMRK-1 mRNA expression levels in MI female mice when compared with non-exposed and CS-exposed (non-MI) female groups was also observed. No significant change in NMRK-1 mRNA expression levels in all male mice groups, however, was seen. Cross-sex analysis revealed a substantial increase in renal NMRK-1 mRNA expression levels of MI and CS-exposed MI female mice when compared with the relative male groups. Figure 6C shows a significant decrease in renal SIRT-1 mRNA expression levels in CS-exposed, MI, and CS-exposed MI male mice when compared with the non-exposed group. CS-exposed female mice exhibited a marked decrease in renal SIRT-1 mRNA expression levels when compared with non-exposed mice. Conversely, MI and CS-exposed MI female mice showed a significant increase in renal SIRT-1 mRNA expression levels when compared with the CS-exposed group. Cross-sex analysis revealed a significant increase in renal SIRT-1 mRNA expression levels in MI and CS-exposed MI female mice when compared with the relative male mice. Figure 6D shows a significant decrease in renal SIRT-3 mRNA expression levels in CS-exposed (non-MI), MI, and CS-exposed MI mice of both sexes when compared with the non-exposed groups. Both MI and CS-exposed MI female mice exhibited a marked increase in renal SIRT-3 mRNA expression levels when compared with CS-exposed (non-MI) female mice. Cross-sex analysis revealed a substantial increase in renal SIRT-3 mRNA expression levels in MI and CS-exposed MI female mice when compared with the relative male groups. Figure 6E shows a significant increase in renal PARP-1 mRNA expression levels in MI male mice when compared with non-exposed and CS-exposed (non-MI) groups. CS-exposed MI-male mice experienced a marked decrease in renal PARP-1 mRNA expression levels when compared with MI mice. No significant change in all female mice, however, was observed. Cross-sex analysis revealed a substantial increase in renal PARP-1 mRNA expression levels in MI male mice when compared with the relative female group. As for total renal NAD levels, Figure 6F reveals a significant decrease in total renal NAD levels in CS-exposed MI males compared with non-exposed, CS-exposed (non-MI), and MI mice. A marked decrease in total renal NAD levels in CS-exposed MI female mice when compared with the CS-exposed (non-MI) group was also observed. No significant change in total renal NAD levels in CS-exposed (non-MI) groups when compared with non-exposed mice in both sexes was observed. MI male mice experienced a substantial decrease in total renal NAD levels when compared with the non-exposed group, whereas MI female mice showed a marked decrease in total renal NAD levels when compared with the CS-exposed (non-MI) group. No significant difference between control male and female mice was observed.

## Discussion

Findings of the present study are novel and of upmost importance. Numerous studies reported a strong association between CS, CVDs, and adverse kidney events [3,21,35,36]. In the present study, mice were primarily exposed to CS for 2 weeks, followed by the induction of MI by ligating the LAD artery, then exposed to CS for 1 week after in order to investigate the effect of CS on kidneys damage post-MI. Hemodynamic analysis revealed a significant decrease in EF in CS-exposed MI mice of both sexes, being more pronounced in males. CO, however, compensated for EF differences between sexes and comparably decreased in CS-exposed MI male and female mice. This finding indicates that the observed sexual dimorphism in kidney damage is not due to differences in kidney perfusion between both sexes, at least at the systemic level. Similarly, inflammation was at its lowest levels in CS-exposed groups 7 days post-MI, with plasma TNF-α being below the detectable limit (<5pg/ml) in both sexes, ruling out therefore any potential positive correlation between systemic inflammation and kidney damage. These findings are in accordance with previous investigations highlighting that pro-inflammatory cytokines such as TNF-α are released 4–6 weeks following CS exposure [3,37]. Additionally, TNF-α secretion has been shown to be markedly enhanced in the early inflammatory phase post-MI and down-regulated 24–72 h thereafter [38,39].

With the absence of pronounced differences between CS-exposed MI mice of both sexes in cardiac hemodynamic parameters and systemic inflammation, sex-based kidney damage discrepancy was tracked down to the kidney molecular and structural levels. Histologically, our findings revealed increased renal morphological alterations in CS-exposed MI mice of both sexes but to a greater extent in male mice. For instance, glomerular retraction, renal fibrosis, and PCT-cross-sectional area markedly increased in CS-exposed male mice post-MI when compared with their relative female group. These findings correlate with a study done by Kobeissy et al. demonstrating that PCT swelling and glomerular retraction, markedly increased in CS-exposed male mice 7 post-MI [40]. Additionally, it has been reported that renal interstitial fibrosis significantly increased following both CS exposure and MI [41,42]. Our findings were further fortified with the pro-fibrotic markers CTGF and α-SMA. Both renal CTGF and α-SMA mRNA expression levels substantially increased in CS-exposed MI male mice, only. Multiple studies demonstrated a pivotal role of CTGF in enhanced kidney fibrosis. For instance, Riser et al. reported increased fibrosis in mesangial cells exposed to recombinant CTGF [43] Additionally, a clinical study has reported a strong correlation between increased plasma CTGF levels and decreased glomerular filtration rate, subsequently enhanced kidney disease progression and development [44]. Similarly, α-SMA, the hallmark of fibroblast differentiation into myofibroblast, has been reported to increase in the interstitium and glomeruli, resulting therefore in enhanced fibrogenesis in the kidneys [45,46]. Coherently, IF data showed accumulation of α-SMA in the peritubular space in the kidneys of MI and CS-exposed MI male mice, only. MI/CS-induced kidney damage is majorly attributed to substantial ROS production [37,47,48]. Our study revealed a comparable increase in renal ROS generation in CS-exposed MI mice of both sexes. This outcome indicates that the protection observed in CS-exposed female mice post-MI is not due to the attenuation of ROS production, but rather due to a mechanism downstream of ROS-induced kidney damage [49,50]. Prolonged ROS production due to exogenous or endogenous stimuli is tightly linked to mitochondrial dysfunction, energy imbalance, and cell death [47,51,52]. Our TUNEL assay showed a comparable increase in glomerular DNA fragmentation in CS-exposed MI mice of both sexes, a finding consistent with previous studies supporting the glomerular cell death stimulating effects of CS and MI [53,54]. DNA fragmentation, however, is a common feature of both apoptosis and necrosis. To further characterize the type of cell death involved, kidney NAD depletion was assessed as a marker of cell death by necrosis and energy failure [55]. NAD levels significantly decreased in CS-exposed MI male mice only when compared with the MI group, supporting a potential necrotic type of cell death linked to mitochondrial dysfunction. [56,57]. Multiple studies report a strong correlation between decreased NAD levels and enhanced kidney injury [56,57]. NAD in the kidney is mainly produced via the amidated pathway through two enzymes NAMPT and NMRK-1. NAMPT converts nicotinamide (NAM) into nicotinamide mononucleotide (NMN), whereas NMRK-1 uses nicotinamide riboside (NR) as a precursor to generate NMN. NMN is then converted trough nicotinamide mononucleotide adenyltransferase (NMNAT) into NAD [57]. NAD has recently emerged as an important moiety used by different enzymes involved in the regulation of energy metabolism and mitochondria biogenesis including SIRT-1 and SIRT-3 as well as enzymes implicated in DNA repair such as PARP-1 [58]. Our findings revealed that unlike CS-exposed MI male mice, CS-exposed MI female mice showed a significant increase in NAD^+^ biosynthetic salvage enzymes expression, including NMRK-1 and NAMPT. Veer et al. and Piallai et al. demonstrated that enhanced NAMPT expression level extends cellular life span through inducing SIRT-1 mediated mitochondrial protein deacetylation [59,60]. Enhanced NAMPT levels subsequently induce SIRT1 expression and activity through replenishing NAD levels, and improving consequently mitochondrial bioenergetic function and cellular integrity [61–64]. In the present study, both NAMPT and SIRT1 expression levels significantly increased in CS-exposed female mice post-MI when compared with their relative male group and are in line with previous findings [63–65]. Of note, SIRT-1 has been shown to protect against ROS-induced injury through the activation of class O of forkhead box (FOXO) and peroxisome proliferator-activated receptor gamma coactivator 1-alpha (PGC-1α), mitochondrial transcription factors, and subsequently enhancing the expression of mitochondrial targeted antioxidant enzymes [66]. However, NAD and SIRT1 levels were markedly decreased in the CS-exposed MI male group when compared with their relative female group. SIRT-3 substantially decreased in CS-exposed MI mice of both sexes, being more pronounced in male mice. Our data are consistent with multiple studies highlighting the link between kidney injury and decreased SIRT-3 mRNA expression levels. SIRT-3 activity significantly decreased in an animal model of diabetic nephropathy resulting in enhanced mesangial cells hypertrophy [67]. Similarly decreased SIRT-3 mRNA levels and increased mitochondrial protein hyperacetylation are reported in an animal model of cystatin-induced AKI [68]. PARP-1, on the other hand, substantially decreased in CS-exposed MI male mice when compared with the MI group, while remained unchanged in all female mice groups. It has been demonstrated that PARP-1 is activated in response to ROS overproduction, decreasing therefore DNA damage, in line with our outcomes indicating enhanced kidney damage in CS-exposed MI male mice [58]. The differential expression in NAMPT, NMRK-1, SIRT-1, and SIRT-3 levels along with the comparable increase in ROS levels in the kidneys of CS-exposed MI mice of both sexes, could explain the sex-biased difference in kidney protection downstream of ROS injury. [69].

## Conclusion and future directions

The importance of the findings revealed in the present study is twofold: (1) CS exacerbated cardiac systolic dysfunction following MI in both sexes; (2) kidney damage was more pronounced in CS-exposed male mice post-MI when compared with the relative female group; and both sexes were characterized by renal morphological alterations, being more pronounced in CS-exposed MI male mice. The observed sex-based kidney damage in the present study seems to be independent of cardiac dysfunction and systemic inflammation, but triggered downstream ROS generation given that both CS-exposed MI male and female mice exhibited similar levels of renal ROS production. For instance, a marked decrease in renal NAD levels and mitochondrial bioenergetic enzymes along with a significant increase in pro-fibrotic markers were observed in CS-exposed MI males, suggesting, potentially enhanced mitochondrial dysfunction, necrotic cell death, and fibrosis when compared with the relative female group. The protection observed in females is potentially linked to the well-known protective impact of estrogen on the cardiovascular system in general and the kidneys in particular. For instance, estrogen receptor-α is expressed in podocytes and exerts renoprotective effects against experimentally induced cell death [70]. Additionally, both tubular injury and macrophage infiltration increased substantially in female mice following estrogen depletion [71], whereas the administration of E2 to aging Dahl salt sensitive rat attenuates tubulointerstitial fibrosis and glomerulosclerosis [72,73]. Mechanistically, E2 seems to enhance expression of both SIRT-1 and NAMPT in response to prolonged ROS production, resulting subsequently in decreased cell death through the inhibition of the pro-apoptotic biomarker caspase 3 [74,75]. Future experiments tailored to detect the protein expression levels of NAD biosynthetic and dependent enzymes and the downstream affected pathways are warranted. Determining the relationship between NAD depletion and the associated apoptosis/necrosis and inflammation may also be needed. Additionally, using SIRT1 and SIRT-3 knock out male mice may be needed to detect whether the observed exacerbated kidney damage in CS-exposed MI male mice is due to decreased SIRT-1 and SIRT-3 levels, consequently potential enhanced mitochondria dysfunction. Last but not least, conducting the same experiments on ovariectomized CS-exposed female mice post-MI is necessary to reveal whether estrogen is primarily or partially involved in the observed protective effects.

## Abbreviations

AKI: acute kidney injury
CO: cardiac output
CS: cigarette smoking
CTGF: connective tissue growth factor
CVD: cardiovascular disease
DHE: dihydroethidium
EF: ejection fraction
GAPDH: glyceraldehyde-3-phosphate dehydrogenase
IF: immunofluorescence
LAD: left anterior descending
MI: myocardial infarction
MTC: Masson’s trichrome
NAMPT: nicotinamide phosphoribosyltransferase
NMRK-1: nicotinamide riboside-1
PAS: periodic acid Schiff
PCT: proximal convoluted tubule
ROS: reactive oxygen species
TBS: tris-buffered saline
α-SMA: alpha-smooth muscle actin
